# An “off-the-shelf” CD2 universal CAR-T therapy for T-cell malignancies

**DOI:** 10.1038/s41375-023-02039-z

**Published:** 2023-10-05

**Authors:** Jingyu Xiang, Jessica M. Devenport, Alun J. Carter, Karl W. Staser, Miriam Y. Kim, Julie O’ Neal, Julie K. Ritchey, Michael P. Rettig, Feng Gao, Garrett Rettig, Rolf Turk, Byung Ha Lee, Matthew L. Cooper, John F. DiPersio

**Affiliations:** 1 Division of Oncology, Department of Medicine, Washington University in St. Louis, St. Louis, MO USA; 2 Division of Dermatology, Department of Medicine, Washington University in St. Louis, St. Louis, MO USA; 3 Division of Public Health Sciences, Department of Surgery, Washington University in St. Louis, St. Louis, MO USA; 4 Integrated DNA Technologies, Coralville, IA USA; 5 NeoImmuneTech, Inc., Rockville, MD USA

**Keywords:** Cancer immunotherapy, Immunotherapy

## Abstract

T-cell malignancies are associated with frequent relapse and high morbidity, which is partly due to the lack of effective or targeted treatment options. To broaden the use of CAR-T cells in pan T-cell malignancies, we developed an allogeneic “universal” CD2-targeting CAR-T cell (UCART2), in which the CD2 antigen is deleted to prevent fratricide, and the T-cell receptor is removed to prevent GvHD. UCART2 demonstrated efficacy against T-ALL and CTCL and prolonged the survival of tumor-engrafted NSG mice in vivo. To evaluate the impact of CD2 on CAR-T function, we generated CD19 CAR-T cells (UCART19) with or without CD2 deletion, single-cell secretome analysis revealed that CD2 deletion in UCART19 reduced frequencies of the effector cytokines (Granzyme-B and IFN-γ). We also observed that UCART19ΔCD2 had reduced anti-tumor efficacy compared to UCART19 in a CD19+NALM6 xenograft model. Of note is that the reduced efficacy resulting from CD2 deletion was reversed when combined with rhIL-7-hyFc, a long-acting recombinant human interleukin-7. Treatment with rhIL-7-hyFc prolonged UCART2 persistence and increased survival in both the tumor re-challenge model and primary patient T-ALL model in vivo. Together, these data suggest that allogeneic fratricide-resistant UCART2, in combination with rhIL-7-hyFc, could be a suitable approach for treating T-cell malignancies.

## Introduction

T-cell acute lymphoblastic leukemia (T-ALL) and cutaneous T-cell lymphoma (CTCL) represent aggressive groups of T-cell malignancies associated with frequent relapse and high morbidity in children and adults [1]. While CD19 CAR-T is successful in treating patients with B-cell malignancies, the utilization of CAR-T cell therapy is still largely restricted due to fratricide, risk of Graft-versus-Host Disease (GvHD), and immune escape, as it is technically challenging to purge all the malignant T cells from an autologous product [2].

Our group and others have independently demonstrated CD7 as a promising therapeutic target for T-cell malignancies [3–6]. Using the CRISPR/Cas9 gene editing approach, we have previously developed an allogeneic “universal” CD7-targeting CAR-T (UCART7) that effectively targets CD7-positive T-cell malignancies. Simultaneous CRISPR/Cas9 deletion of TRAC and CD7 prevents CAR-T cell fratricide and permits the safe use of allogeneic T cells by eliminating the potential for Graft-versus-Host Disease (GvHD) [3]. However, CD7 is classically downregulated or absent in some T-cell malignancies such as adult T-cell leukemia/lymphoma (ATL) and Sezary syndrome (SS) [7, 8]. While most patients with CD7-positive T-cell malignancies showed good responses to the CD7-targeting CAR-T therapy, relapse of CD7-negative disease has been reported in recent clinical trials [6, 9, 10]. In contrast, CD2 is expressed on >99% of T-cells and downregulates at a lower frequency than other pan-T cell markers in cancer cells, providing an attractive therapeutic target [11, 12].

CD2 is a transmembrane glycoprotein restricted to hematopoietic cells with high expression in NK cells and T cells, including early T cell progenitors. CD2 acts as a co-stimulatory receptor with roles in thymocyte development, actin cytoskeleton rearrangement, and cellular signaling at the immunological synapse [13]. In conjunction with its ligands CD58 (in human) and CD48 (in mice), CD2 co-stimulation plays an important role in T cell activation and TCR signaling [14, 15]. CD2 is expressed in a wide variety of T-cell malignancies, including T-ALL, SS (Sezary Syndrome), peripheral T-cell malignancies, and adult T-cell leukemia/lymphoma (ATL). Therefore, we hypothesized that CD2 and TRAC could be deleted from T-cells of allogeneic donors by CRISPR/Cas9, then transduced with a CD2 targeting CAR (CAR2-CD28-CD3ζ) to effectively kill T-cell malignancies without resulting in GvHD or CAR-T cells fratricide. Loss of CD2 may hamper T cell cytotoxicity but is unavoidable when using this as a target for CAR-T cell therapy. We further hypothesized that loss in the efficacy of CAR-T function resulting from CD2 deletion could be compensated for by co-treatment with rhIL-7-hyFc (recombinant human interleukin 7 fused with hybrid Fc), a stable, long-acting, homo-dimeric interleukin-7 (IL-7) molecule that effectively enhances the expansion, efficacy, and persistence of CAR-T cells [16, 17]. The data presented here demonstrate the feasibility of generating UCART2 and provide evidence that supports the anti-tumor efficacy of UCART2 for the treatment of mature T-cell malignancies. Furthermore, in vivo administration of rhIL-7-hyFc in pre-clinical models elevated UCART2 into a curative therapy for treating T-cell malignancies.

## Materials and methods

Detailed materials and methods are described in Supplemental Methods.

## Results

### CD2^Δ^ TRAC^Δ^ CART2 (UCART2) kills CTCL and T-ALL in vitro without fratricide

**Fig. 1:**
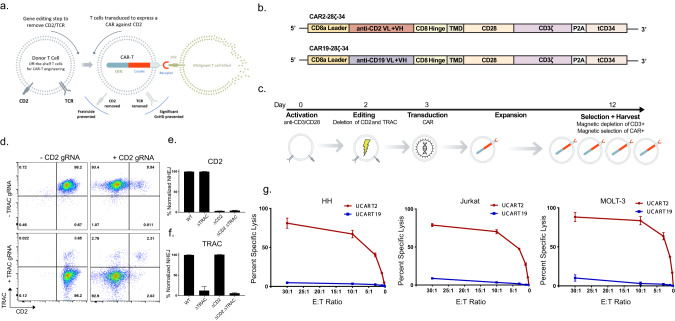
UCART2 kills T-ALL and CTCL in vitro. **a** Schematic of UCART2 design. **b** Schematic of CAR2-28ζ-34 and CAR19-28ζ-34 CAR constructs. **c** Timeline of UCART2 production with CRISPR/Cas9 gene editing. T cells were cultured in Xcyte media supplemented with 50 U/mL IL-2 and 10 ng/ml IL-15 and activated with anti-CD3/CD28 beads (bead to cell ratio 3:1) for two days followed by T-cell transduction of either CD2 or CD19 CAR construct. Transduced T cells were expanded for 9 days, followed by CD3+ depletion and hCD34 enrichment. Efficiencies of multiplex CRISPR/Cas9 gene editing of CD2 and TRAC were assessed by **d**. flow cytometry (CD2 vs. TRAC), or **e,** **f**. targeted deep sequencing of CD2 or TRAC. % NHEJ was determined as a percentage of sequencing reads with indels relative to WT cells. **g** In vitro killing assay. UCART2 or UCART19 cells were cultured with ^51^Cr-labeled HH, Jurkat, or Molt-3 cells at various E: T ratios for 4 h. Specific lysis was calculated based on released ^51^Cr in the culture medium.

We previously developed an allogeneic UCART7 that effectively targets CD7 positive T-cell malignancies [3]. To test whether CD2 could be an effective therapeutic target for T-cell malignancies, we generated UCART2 (CAR2-28ζ-34 with CD2^Δ^ TRAC^Δ^), a gene-edited CAR-T in which the CD2 antigen is deleted to prevent fratricide and the T-cell receptor (TRAC) is removed to prevent GvHD (Fig. 1a–c). Seven days post CRISPR/Cas9 mediated deletion of CD2 and TRAC in primary T-cells, surface expression of CD2 and TRAC were examined by flow cytometry, which showed > 95% double deletion of CD2 and TRAC in samples co-electroporated with both CD2 and TRAC gRNA (Fig. 1d). In line with this data, targeted deep sequencing also confirmed that the loss of expression resulted from frameshift Indels in 95.7% (SD±0.96%) of *CD2* reads (Fig. 1e) and 94.2% (SD±1.59%) of *TRAC* (Fig. 1f). Characterizing the off-target nuclease activity profile is essential for proving the safety of gene-edited CAR-T cells. Therefore, we used Guide-Seq to assess off-target sites of CRISPR/Cas9 gene editing in a genome-wide unbiased fashion (Supplemental Fig. 1). Across all three replicates, on-target reads (*CD2* and *TRAC* reads per total reads) represented between 84.2% and 89% of all capture sequences. The *TRAC* gRNA had a clean off-target profile with minimal off-target events detected in three non-coding regions. Ten off-target sites with CD2 gRNA were identified, with six sites identified within intergenic regions of genomic DNA and the remaining four sites within the exons of genes (*HEXB*, *CALR*, *KIF21B*, and *MUC4*). However, the off-target reads within these four genes were significantly lower than the on-target CD2 reads.

Next, we tested the ability of UCART2 to specifically kill CD2+T-ALL and CTCL cell lines using a Cr^51^ release cytotoxicity assay. Cr^51^ labeled CD2+ target cells (HH, Jurkat, and MOLT-3) were co-cultured with UCART2 or UCART19 at various E: T ratios (30:1 to 0.3:1) for 4 h, and the percentage of target-specific lysis was determined by released Cr^51^ (Fig. 1g). UCART2 demonstrated potent cytotoxicity against all three CD2+ tumor cell lines in vitro (*p* < 0.0001). In CTCL cell line HH, 65% of target-specific cytolysis was achieved at the 10:1 E: T ratio. Similarly, the T-ALL cell lines, Jurkat and MOLT-3, were equally sensitive to UCART2-mediated killing with >70% and >80% target-specific cytolysis at the 10:1 ratio, respectively, over 4 h. In contrast, target-specific killing was not observed with the non-targeting UCART19 control.

### UCART2 prolongs survival and reduces tumor burden in vivo

**Fig. 2:**
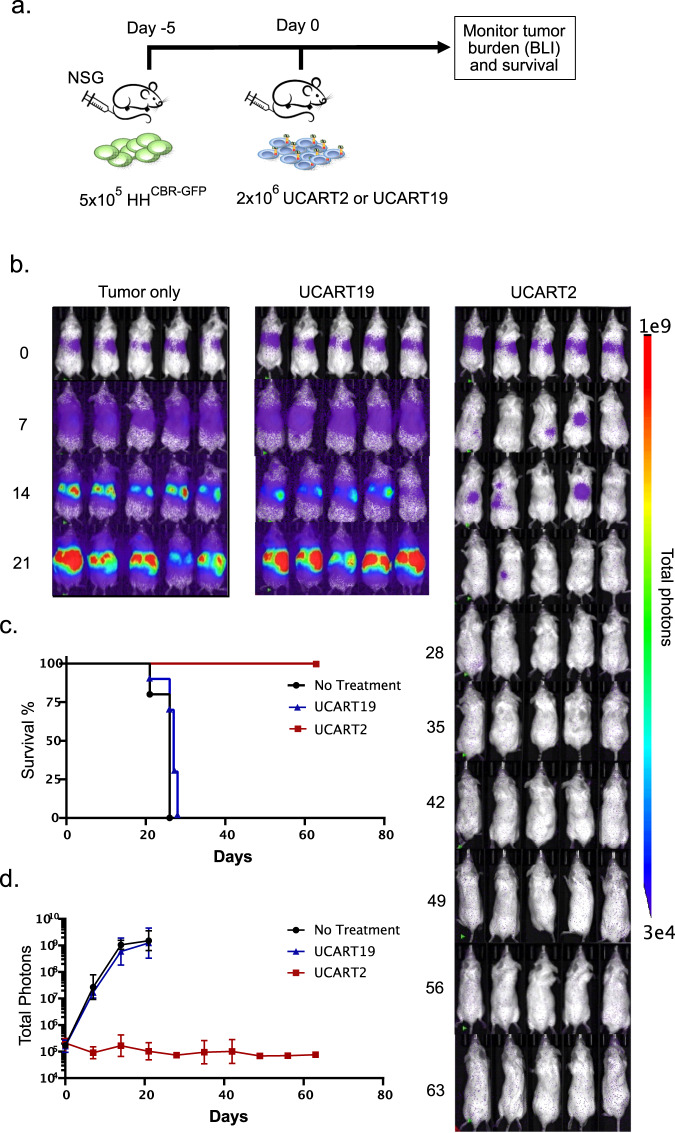
In vivo efficacy of UCART2 in a xenogeneic model of CTCL. **a** Schema of the xenogeneic mouse model of CTCL. NSG mice were injected with 5 × 10^5^ HH^CBR-GFP^ cells on day -5, then infused with 2 × 10^6^ UCART2 or UCART19 on day 0. **b,** **c** tumor burden was assessed with BLI weekly (*n* = 5 per group). **d** Kaplan–Meier survival curve of mice treated UCART19, UCART2, or untreated control. Median survival: untreated mice (26 days), UCART19 treated mice (27 days), UCART2 treated (no death at the end of the experiment on day 65, *p* < = 0.0001).

To test the in vivo efficacy of UCART2, 5 × 10^5^ CD2+CTCL cell line HH^CBR-GFP^ was inoculated in NSG mice by I.V. injection (day -5), followed by I.V. infusion of 2 × 10^6^ UCART2 or UCART19 on day 0 (Fig. 2a). Tumor burden was monitored weekly by BLI (bioluminescence imaging, Fig. 2b). In contrast to the mice injected with tumor-only or UCART19 cells, UCART2 significantly prolonged the survival of the CTCL tumor-bearing mice (*p* < 0.0001). Remarkably, while the median survival of both control groups (tumor only and UCART19) was 27 days, all the UCART2-treated mice were alive by the end of the experiment (day +63; Fig. 2c). Similar to our previous findings with UCART7, no signs of GvHD were observed in mice treated with UCART2 or UCART19 (*data not shown*). Consistently, compared to the untreated tumor-only group, BLI also showed significant attenuation of the tumor burden in UCART2-treated animals (*p* < 0.0001) but not in UCART19-treated animals (Fig. 2d). Together, these data demonstrated that UCART2 is effective and specific to kill CD2+HH CTCL cells in vivo.

### CD2 deletion attenuates CAR-T function in vivo

**Fig. 3:**
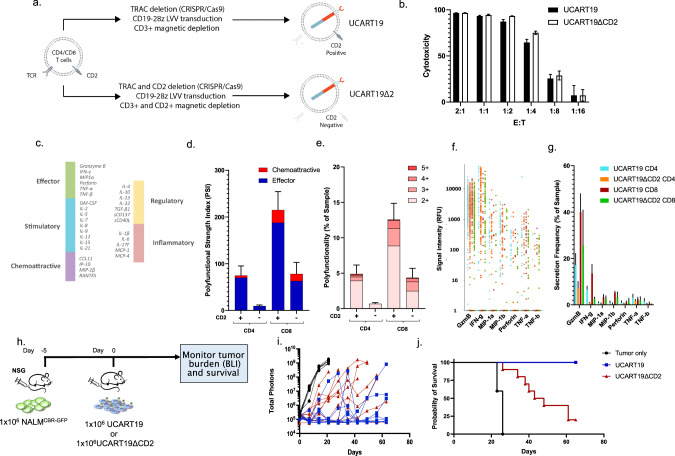
CD2 deletion affects the efficacy of UCART19. **a** Schema of the generation of UCART19 (TRAC-CD2+) and UCART19ΔCD2 (TRAC-CD2-). **b** in vitro killing efficacy of UCART19 and U CART19ΔCD2. UCART19 and UCART19ΔCD2 were cultured with Nalm6 ^CBRGFP^ CD19+ targets at various E: T ratios for 16 h and target-specific killing was measured by luciferase activity. **c** Single-cell cytokine analysis using the IsoCode assay. UCART19 and UCART19ΔCD2 were incubated with B-cell lymphoma cell line Ramos at an E: T 1:2 for 20 h prior to loading purified CAR-T populations (CD19 depleted and CD34-affinity purified) onto the IsoCode chip. **d** Polyfunctionality and **e** Polyfunctional strength index (PSI) of the CD4 or CD8 subpopulation from UCART19 (CD2 +) or UCART19ΔCD2 (CD2-). **f** The signal strength of key cytokines driving polyfunctionality (GMZB, IFN-γ, MIP-1α, MIP-1β, Perforin, TNF-α, and TNF-β) was not affected by CD2 deletion. **g** The frequency of cells secreting Granzyme (Gzmb), IFN-γ, MIP1α, MIP1-β, Perforin, TNF-α, and TNF-β in the CD4 or CD8 subpopulation from UCART19 or UCART19ΔCD2. **h** Schema of the xenogeneic mouse model of CD19 + B cell acute lymphoblastic leukemia: 1 × 10^6^ NALM6^CBR-GFP^ cells were I.V. inoculated into NSG mice on day -5, followed by infusion of 1 × 10^6^ UCART19 or UCART19ΔCD2 on day 0 (*n* = 10 per group). **i** Tumor burden as determined by BLI. **j** Kaplan–Meier survival curve. Median survival: untreated mice, 26 days, mice treated with UCART19 ΔCD2, 45.5 days, all mice treated with UCART19 were alive at day +65.

CD2 plays an important role in T cell activation and the formation and organization of the immunological synapse [13, 18]. A recent report suggests that loss of CD2 co-stimulatory receptor CD58 limits the durable CD19 CAR response in large B cell lymphoma [19]. Therefore, we set out to evaluate the potential impact of CD2 deletion on CAR-T function by generating the UCART19ΔCD2 cells, in which CD2 and TRAC were genetically deleted, as compared to UCART19, in which only TRAC was deleted while CD2 remained unmodified (Fig. 3a). Deletion of CD2 in UCART19 had little impact on the CD4:CD8 ratio (Supplemental Fig. 2a) or immune phenotype within the CD4+ or CD8+ subset (Supplemental Fig. 2b). We first tested the in vitro efficacy of UCART19 or UCART19ΔCD2 in a 24 h flow-based killing assay by co-culture of CAR-T cells with CD19+NALM6^CBR-GFP^ targets. Deletion of CD2 had no immediate deleterious effect on UCART19 function in vitro, maintaining similar cytotoxicity against CD19+ targets in this 24 h killing assay (Fig. 3b). To further characterize the impact of CD2 deletion in CAR-T cells, we performed single-cell secretome analyses on target-stimulated UCART19 and UCART19ΔCD2 using the Isolight platform (Isoplexis). The Isolight assay enables the detection and quantification of 32 secreted cytokines from individual CAR-T cells to define polyfunctional strength index (PSI), a quantitative parameter of T cell functionality (Fig. 3c) [20]. Twenty hr after co-culture with the target cells (E:T = 1:2), CAR-T cells were isolated for the Isolight assay. Single-cell secretome analysis of target-stimulated UCART19 (CD2 +) and UCART19ΔCD2 (CD2-) cells revealed that deletion of CD2 in UCART19 reduced the PSI in both the CD4+ and CD8+ populations (Fig. 3d). This reduction in PSI was driven by a decrease in the percentage of UCART19 secreting two or more cytokines (Fig. 3e) and not a reduction in the intensity of cytokine secretion. Similar signal intensities were observed for Granzyme B (GzmB), IFN-γ, MIP-1α, MIP-1β, perforin, TNF-α, and TNF-β between UCART19 and UCART19ΔCD2 (Fig. 3f). In contrast, reduced secretion frequencies of the effector cytokines (GzmB and IFN-γ) were observed in both the CD4+ and CD8+ population when CD2 was deleted in UCART19 (Fig. 3g), suggesting that the CD2:CD58 axis may play a key role in CAR-T co-stimulation.

Next, to test whether CD2 deletion in UCART19 would attenuate CAR-T efficacy in vivo we used the CD19+NALM6 xenogeneic model. We inoculated 1 × 10^6^ NALM6^CBR-GFP^ I.V. into NSG mice on day -5, followed by I.V. infusion of either 1 × 10^6^ UCART19 or UCART19ΔCD2 on day 0. Tumor burden was assessed weekly using BLI (Fig. 3h). Both UCART19 and UCART19ΔCD2 treatment significantly decreased tumor burden in vivo compared to the untreated control animals (*p* < 0.001; Fig. 3i). Compared to mice treated with UCART19, UCART19ΔCD2 treated mice demonstrated reduced suppression of tumor growth in vivo (Fig. 3i). In addition, while both UCART19 and UCART19ΔCD2 significantly prolonged survival (Log-rank test: *p* < 0.0001; *p* = 0.0005, respectively), deletion of CD2 in UCART19 significantly shortened the survival in vivo (UCART19 vs. UCART19ΔCD2, *p* = 0.0003) (Fig. 3j). Taken together, these data suggest that CD2 deletion attenuates the efficacy of CAR-T cells in vivo.

### rhIL-7-hyFc enhances the efficacy of UCART2

**Fig. 4:**
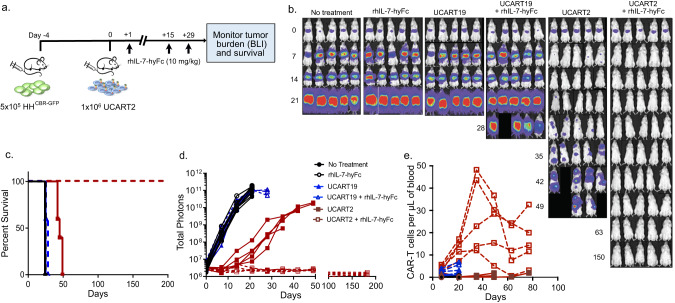
In vivo efficacy of UCART2 in combination with rhIL-7-hyFc. **a** Schema of the xenogeneic mouse model of CTCL. NSG mice were injected with 5 × 10^5^ HH^CBR-GFP^ cells on day -4, then infused with 1 × 10^6^ UCART2 or UCART19 on day 0. 10 mg/kg of rhIL-7-hyFc was delivered subcutaneously on day 1, day 15, and day 29. **b** tumor burden was measured weekly by BLI. **c** Kaplan–Meier survival curve of mice treated with UCART19, UCART2, UCART19+rhIL-7-hyFc, and UCART2+rhIL-7-hyFc. Median survival: untreated mice, mice treated with NT-17 alone, and mice treated with UCART19 were 25 days, mice treated with UCART19 + NT-17 was 26.8 days, mice treated with UCART2 alone was 45.4 days, mice treated with UCART2+rhIL-7-hyFc were all alive at 200 days when the experiment was ended. **d** Tumor burden as determined by BLI. **e** Flow cytometry analysis was performed bi-weekly to assess circulating CAR-T cells present per µl of peripheral blood. CAR-T population determined by hCD45+, 7aad-, hCD34+ cells. *P* values < 0.05 considered significant, *p ≤ 0.05, **p ≤ 0.01, ***p ≤ 0.001, ****p ≤ 0.0001. **d** BLI images normalized to a color gradient scale.

To overcome the reduction of CAR-T efficacy resulting from CD2 deletion, we evaluated whether combining with the long-lasting human IL-7, rhIL-7-hyFc, could enhance the efficacy of UCART2. We have previously demonstrated that rhIL-7-hyFc can enhance the expansion persistence and efficacy of UCART19 and UCART33 in vivo [16, 17]. We hypothesized that UCART2 efficacy could be further improved when combined with rhIL-7-hyFc. We first test this hypothesis in a CAR-T stress model using the serial replating assay: UCART2 cells were re-challenged with CD2+ target cells (Jurkat^CBR/GFP^) every 2–3 days at E:T ratio of 2:1-1:1, CAR-T expansion and CAR-T effector function were monitored. While the UCART2 alone became dysfunctional and lost its killing efficacy in vitro after repeated antigen exposure, rhIL-7-hyFc supplementation significantly enhanced the expansion and anti-tumor activity of UCART2 in vitro (Supplemental Fig. 3). To test this hypothesis in vivo, we inoculated 5 × 10^5^ HH^CBR-GFP^ CTCL cells I.V. into NSG mice (day -5), followed by UCART2 or UCART19 treatment on day 0. Due to the high efficacy of UCART2 observed in this model (Fig. 2), mice were treated with a suboptimal dose (1 × 10^6^) of UCART2 or UCART19 to allow the observation of the effect from rhIL-7-hyFc. Mice were treated with rhIL-7-hyFc (10 mg/kg) or vehicle subcutaneously on Days +1, +15, and +29 following UCART infusion. The tumor burden was monitored weekly by BLI (Fig. 4a). As expected, UCART2 significantly reduced tumor burden, as measured by BLI (Fig. 4b). In vivo administration of rhIL-7-hyFc converted UCART2 into a curative therapy, reducing the tumor burden back to baseline by day +20 and no relapse was observed during the full study duration of 200 days (Fig. 4c). In line with this finding, while the median survival time was 25 days in non-targeting UCART19 controls and 45 days in UCART2 alone, all the mice from the UCART2/rhIL-7-hyFc group were alive by the end of the study (day 200) (Fig. 4d). These data demonstrate that rhIL-7-hyFc dramatically enhances the efficacy of UCART2 and prolongs survival in vivo. Of note is that the administration of rhIL-7-hyFc significantly increased the numbers of UCART2 and enhanced the persistence of UCART2 in vivo (Fig. 4e), largely contributing to the enhanced survival observed in Fig. 4c. In spite of UCART2 lacking endogenous CD2, rhIL-7-hyFc was able to overcome the potential functional deficit of these UCART2 in vivo. In line with this data, using the serial replating assay in vitro, we also observed that rhIL-7-hyFc was able to rescue the reduced effector function of CAR-T cells due to CD2 loss in a CART19 model (Supplemental Fig. 4).

### rhIL-7-hyFc prolongs UCART2 persistence in vivo and overcomes tumor re-challenge

**Fig. 5:**
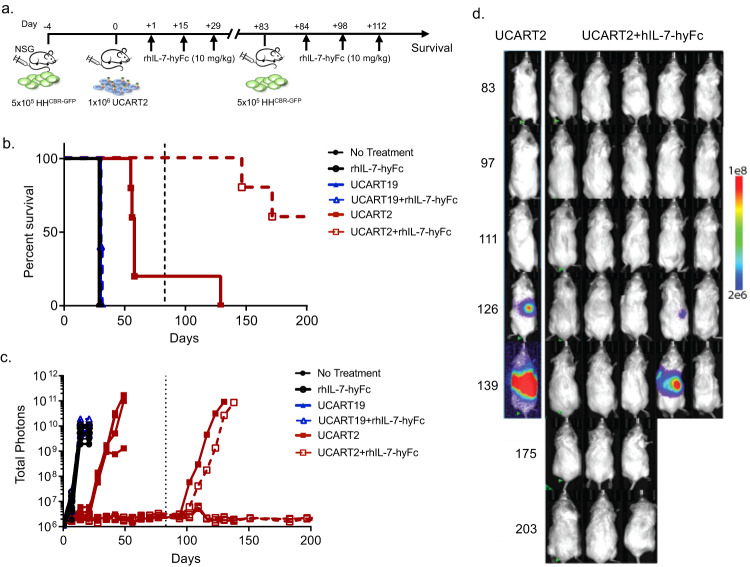
rhIL-7-hyFc prolongs UCART2 persistence in vivo and overcomes tumor re-challenge. **a** Schema of the CTCL re-challenge mouse model. NSG mice were injected with 5 × 10^5^ HH^CBR-GFP^ cells on day -4, then infused with 1 × 10^6^ UCART2 or UCART19 on day 0. 10 mg/kg of rhIL-7-hyFc was delivered subcutaneously on day +1, day +15, and day +29. Surviving mice were re-challenged with 5 × 10^5^ HH^CBR-GFP^ cells on day +83. 10 mg/kg of rhIL-7-hyFc was delivered subcutaneously on days +84, +98, and 112. **b** Kaplan–Meier survival curve. Mice receiving UCART. UCART19 Vs. UCART2, Median survival 30 days Vs. 58 days *p* = 0.0027. UCART19+rhIL-7-hyFc Vs. UCART2+rhIL-7-hyFc *p* = 0.0023 30 days Vs. 200+ days. **c** Tumor burden as determined by BLI imaging. **d** Normalized BLI images.

We next performed a re-challenge model to address the functional persistence of UCART2 expansion in vivo over time when combined with rhIL-7-hyFc. Using the same HH CTCL model, surviving mice (1 from UCART2 and 5 from the UCART2/rhIL-7-hyFc group) on day +83 were re-challenged with a second inoculation of HH^CBR-GFP^ cells, followed by three doses of rhIL-7-hyFc (Fig. 5a). As expected, UCART19 with or without rhIL-7-hyFc treatment had no effect on survival (median survival: 30 days), while the suboptimal dose of UCART2 significantly prolonged the survival (median survival: 58 days) compared to the tumor-only control (Fig. 5b). Compared to the UCART2 alone treatment, the addition of rhIL-7-hyFc further extended the survival of the animals (median survival: not reached, >200 days). Remarkably, four out of five rhIL-7-hyFc/UCART2 treated mice cleared tumors upon challenge, with three mice surviving the full duration of the experiment (Fig. 5c, d). Together, these data suggest that rhIL-7hyFc propagates the long-term persistence of UCART2 to overcome the tumor re-challenge in vivo.

### UCART2, in combination with rhIL-7-hyFc, kills primary patient-derived T-ALL in vivo

**Fig. 6:**
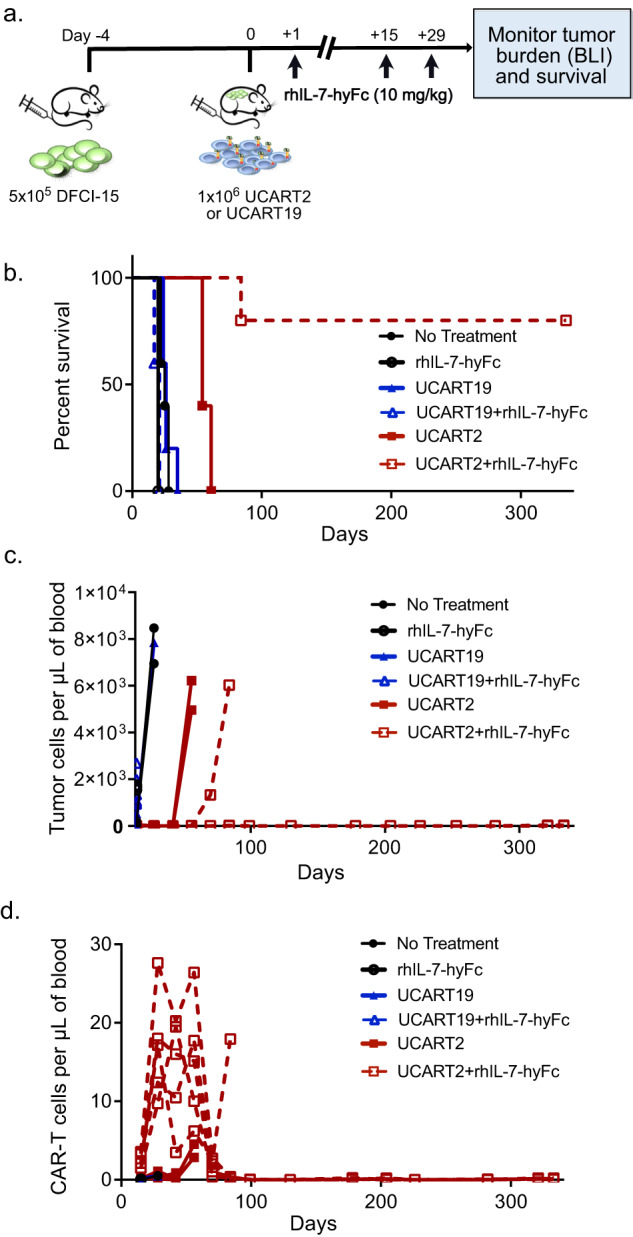
UCART2, in combination with rhIL-7-hyFc, kills primary patient T-ALL in vivo. **a** Schema of the patient-derived T-ALL xenograft model. NSG mice were injected with 5 × 10^5^ DCFI-15 cells on day -12, then infused with 1 × 10^6^ UCART2 or UCART19 on day 0. 10 mg/kg of rhIL-7-hyFc was delivered subcutaneously on day +1, day +15, and day +29. **b** Kaplan–Meier survival curve Median survival: untreated mice 26 days, rhIL-7-hyFc alone - 20 days, UCART19 26 days, UCART19+rhIL-7-hyFc - 21 days, UCART2 - 54 days, mice treated with UCART2+rhIL-7-hyFc 300+ days. UCART19 Vs. UCART2 *p* = 0.0019, UCART19+rhIL-7-hyFc Vs. UCART2+rhIL-7-hyFc, *p* = 0.0031. **c** Flow cytometry analysis was performed by-weekly to assess circulating tumor cells present per µl in peripheral blood. Tumor population was determined as hCD45 +, 7-AAD-, hCD34-, hCD2+ cells. **d**. Flow cytometry analysis was performed bi-weekly to assess circulating CAR-T cells present per µl in peripheral blood. CAR-T population determined by hCD45 +, 7-AAD-, hCD34 +, hCD2- cells.

To test the efficacy of UCART2 against cancer cells from patients, we established a CD2+ primary T-ALL xenograft model. Patient-derived DFCI-15 cells were injected I.V. into NSG mice on day -12 and treatment with UCART2 or UCART19 was administered I.V. on day 0, followed by rhIL-7-hyFc (10 mg/kg) or vehicle on day +1, +15 and +29 (Fig. 6a). Treatment with UCART2 alone doubled the survival of PDX-bearing mice relative to UCART19 (median survival: UCART19, 26 days; UCART2, 54 days, *p* = 0.0019), demonstrating effective antitumor activity in the absence of rhIL-7-hyFc (Fig. 6b). rhIL-7-hyFc treatment, in the absence of CAR-T cell therapy, was trending towards shortened survival in this PDX model (median survival: Vehicle control 26 Days vs. rhIL-7-hyFc 20 Days, *p* > 0.05), suggesting rhIL-7-hyFc itself may have provided a modest proliferative advantage to malignant T cells. The combination treatment of rhIL-7-hyFc and UCART2 dramatically enhanced survival, with 80% of mice surviving beyond 300 days (Fig. 6b). Flow cytometry analysis was performed bi-weekly to assess circulating tumor cells (hCD45+, 7-AAD-, GFP+) and CAR-T cells (hCD45+, 7-AAD-, hCD34+) in the peripheral blood. In comparison with untreated or UCART19 treated animals, UCART2 alone or in combination with rhIL-7-hyFc significantly decreased the systemic tumor burden, as measured by circulating tumor cell counts (Fig. 6c). 4 out of 5 mice treated with UCART2/ rhIL-7-hyFc had no detectable circulating tumor cells in the blood. Concordant with survival and systemic tumor burden, rhIL-7-hyFc enhanced UCART2 expansion and persistence, with a high frequency of circulating CAR-T cells (peak count: 28 cells/μL) detected in the periphery 80 days after CAR-T therapy and 50 days following the last dose of rhIL-7-hyFc treatment (Fig. 6d). Combined, these data suggest that UCART2 alone is effective at killing primary T-ALL tumor cells and that potentiation with rhIL-7-hyFc further improves the expansion and persistence of UCART2, resulting in a dramatic increase in survival with most mice having no evidence of tumor >300 days after treatment with UCART2 and rhIL-7-hyFc.

## Discussion

CAR T-cell therapy has become one of the most promising cancer therapies. It has shown enormous potential for inducing remissions and long-term relapse-free survival in patients with B cell leukemia and lymphoma [21–23]. For T-cell malignancies, however, the shared expression of target antigens on both malignant and healthy T-cells represents a unique set of challenges. Here, we describe UCART2, a novel CD2-targeting allogeneic CAR-T therapy in which the biallelic deletion of CD2 and TRAC prevents fratricide and life-threatening GvHD while effectively killing CD2+ primary human T-ALL and CD2 + T-ALL and CTCL cell lines in vitro and in vivo. Furthermore, our pre-clinical data demonstrates that UCART2, in combination with rhIL-7-hyFc, results in curative and durable therapeutic responses.

For T-cell malignancies, the use of autologous CAR-T therapy is limited due to high manufacture expenses, inconsistent quality, lengthy production time, and the lack of reliable methods to distinguish between effector T cells and malignant T cells. To overcome these limitations, “off-the-shelf” CAR-T cells from allogeneic donors have been increasingly used [24]. Up to date, there are over 50 clinical trials listed on ClinicalTrials.gov evaluating the use of allogeneic CAR-T therapy in hematological malignancies. For T-cell malignancies, CAR-T cells targeting T-cell makers, including CD5 [25, 26], CD7 [6, 27–29], and CD30 [30], are currently being developed and have shown promising responses against T-ALL and lymphoma. Among these target antigens, CD7 is most extensively investigated due to its abundant expression in T-cell malignancies, and multiple studies are underway to examine allogeneic CD7 targeting CAR-T cells [6, 31, 32]. Recent Phase I results from a genetically modified CD7-targeting allogeneic CAR-T cell therapy (RD13-01) demonstrated encouraging efficacy and safety against relapsed/refractory CD7-positive hematological malignancies [6]. We have previously generated allogeneic gene-edited CAR-T targeting CD7 (UCART7) with biallelic deletion of CD7 and the T cell receptor alpha chain (TRAC), allowing for the generation of fratricide-resistant CAR-T targeting CD7+T cell malignancies without the risk of life-threatening GvHD [3]. UCART7 demonstrated pre-clinical safety and efficacy against T-ALL and is currently being tested in a Phase 1/2 clinical trial (WU-CART-007, ClinicalTrials.gov Identifier: NCT04984356). However, as previously stated, CD7 expression could be endogenously absent or downregulated due to the selective pressure by anti-CD7 CAR-T cell therapy in some mature T-cell malignancies, leading to therapeutic resistance and relapse, despite the continued persistence of CAR-T cells [9, 10, 33]. The relapse of CD7- T-cell malignancies, and the absence of CD7 on more mature T-cell malignancies, demonstrates a significant unmet clinical need for developing “off-the-shelf” CAR-T cell therapies against novel T-cell antigens. Targeting CD2, therefore, would allow for the effective targeting of a wide variety of CD7 negative or low expression T cell leukemia and lymphomas such as T-ALL, SS, peripheral T cell malignancies, and adult T cell leukemia/lymphoma (ATL).

Given that CD2 is essential in T-cell activation and T-cell mediated cytotoxicity, we tested the efficacy of UCART19 with and without the deletion of CD2 (UCART19ΔCD2 or UCART19). A study by Majzner et al. has implicated that the CD58 loss in CAR-T resistance in the context of CD19 + B cell malignancies and has suggested that CD58 deletion from the CAR-T cell surface reduced CAR-T activation and reduced cytokine secretion upon CAR-T stimulation [19]. In a more recent study, loss of CD58 was identified from an unbiased genome-wide CRISPR screening approach to confer resistance to CAR-T cell therapy, due to inefficient immunological synapse formation and impaired cytotoxic function of CAR-T cells [34]. In line with this finding, we found that deletion of the counterpart of CD58, CD2 in UCART19 led to a reduction of in vivo efficacy and survival benefit, compared to that seen in UCART19 treated tumor-bearing NSG mice. Furthermore, single-cell cytokine analysis revealed that CD2 deletion decreased the diversity (but not intensity) of secreted cytokines in both CD4+ and CD8 + UCART19ΔCD2 cells. More specifically, UCART19ΔCD2 cells exhibited reduced secretion of GzmB and IFN-γ, consistent with recent findings that the interaction between CD2 and CD58 is critical for T cell activation and TCR signaling [14].

The reduction of CAR-T efficacy resulting from CD2 deletion prompted us to examine whether combination with IL-7 could overcome this defect. IL-7 is integral to the survival of CD8+ naïve and memory T cells, and not surprisingly several clinical trials are in progress to evaluate the therapeutic benefits of IL-7 administration in viral infections (ClinicalTrials.gov Identifier: NCT04501796), lymphopenia (ClinicalTrials.gov Identifiers: NCT05600920, NCT04781309) and cancer (ClinicalTrials.gov Identifiers: NCT05075603). However, IL-7 has a short serum half-life, often requiring frequent and repeated administration [35]. In recent reports, we have defined rhIL-7-hyFc, as a titratable therapeutic strategy to enhance CAR-T cell proliferation, persistence, and tumor killing in vivo [16]. rhIL-7-hyFc consists of a genetically modified human recombinant IL-7 fused to a hybrid neonatal Fc receptor (Hy-Fc), which significantly enhances its stability and half-life in vivo [35, 36]. Encouraged by our recent findings that rhIL-7-hyFc enhances CAR-T cell expansion, persistence and anti-tumor activity, a Phase 1b clinical trial is currently ongoing using rhIL-7-hyFc (efineptakin alfa, NeoImmuneTech, Inc.) following the standard of care CD19 CAR T-cell therapy in patients with Relapsed/Refractory Large B-cell Lymphoma (ClinicalTrials.gov ID: NCT05075603). Our preliminary data suggests that rhIL-7-hyFc treatment following tisagenlecleucel was safe and well-tolerated and did not induce CRS or ICANS [37].

Expanding on our previous data with UCART7 [3], UCART2 in combination with rhIL-7-hyFc led to curative, durable responses in our in vivo model of CTCL, where the mice received sub-optimal UCART2 doses. UCART2 alone doubled the survival times compared with mice receiving the tumor-only or UCART19 plus tumor. When combined with rhIL-7-hyFc, UCART2 dramatically enhanced survival, with 80% of the mice surviving more than 300 days. In the same model of CTCL, we observed the long-term persistence of UCART2 in mice treated with rhIL-7-hyFc by re-challenging mice that had gone into remission. Mice receiving additional doses of rhIL-7-hyFc exhibited a resurgence of circulating UCART2, resulting in prolonged survival in vivo. It is well known that IL-7 aids in the generation and maintenance of T-cell memory, and recent studies have implicated IL-7 in promoting immune cell infiltration and CAR-T survival [38–40]. Our studies are consistent with these findings, suggesting that rhIL-7-hyFc is a viable clinical alternative to traditional IL-7 approaches to enhance the efficacy of CAR-T therapy.

Although IL-7 was known to promote the T-ALL proliferation [41] and mutational activation of IL-7Rα was reported to promote the development of T-ALL [42], we did not observe increased tumor burden or decreased survival with rhIL-7-hyFc treatment alone in either CTCL or T-ALL PDX models. Additional tests with patient-derived samples will be performed in the future to evaluate the effect of rhIL-7-hyFc alone or in combination with UCART2 prior to clinical testing.

Characterizing the off-target nuclease activity profile is essential for demonstrating the safety of gene-edited CAR-T cells. Potentially deleterious deletions/insertions may produce undesirable adverse events when infused into patients. We used Guide-Seq to assess off-target sites of CRISPR/Cas9 gene editing in a genome-wide unbiased fashion. As anticipated, Guide-seq detected a high degree of on-target editing at the *TRAC* and *CD2* loci. Across all three replicates, on-target reads represented between 84.2% and 89% of all capture sequences. The TRAC gRNA had a clean off-target profile with no aberrant editing events occurring within exons and only one off-target locus that was consistently identified across the three replicates. For the CD2 gRNA, four off-target events were detected within the exons of genes include *HEXB* (hexosaminidase subunit beta), *CALR* (calreticulin), *KIF21B* (kinesin family member 21B), and *MUC4* (mucin 4). Of these *HEXB* was the only gene with consistent off-target reads detected. Additionally, disparate homology was observed between target gRNA sequences and GUIDE-seq identified off-target sites, ranging in frequency from 5 to 10 mismatches, suggesting not all sites identified may be bona fide sites of off-target editing. Further characterization of these aberrant editing events would be required before moving UCART2 forward to the clinic.

In summary, we developed a novel allogeneic “off-the-shelf” UCART2 therapy for T-cell malignancies. In preclinical models, UCART2 showed potent responses against T-ALL, CTCL tumor cells, and patient-derived T-ALL xenografts. In combination with rhIL-7-hyFc, UCART2 resulted in durable complete responses in vivo. Since CD2 is one of the surface markers that is the least frequently absent or lost in T-cell malignancies [11], UCART2 may serve as a promising therapy for a wide variety of CD2+T-cell cancers.

### Supplementary information

Supplemental Table 1 & 2

S1

S2

S3

S4

Supplemental Methods

Supplemental figure legend
