# Supplementary material for: An “off-the-shelf” CD2 universal CAR-T therapy for T-cell malignancies
Source: Leukemia. 2023 Oct 5;37(12):2448–56. doi: 10.1038/s41375-023-02039-z (PMC10681896; doi:10.1038/s41375-023-02039-z)
Supplement: Supplementary file 1 — Supplemental Table 1 & 2 [file 41375_2023_2039_MOESM1_ESM.docx]

**Supplemental Table 1.** Guide RNA sequence

| **Name** | **gRNA sequence** |
| --- | --- |
| CD2 | 5’_2′Ome(A(ps)C(ps)A(ps))GCUGACAGGCUCGACACGUUUUAGAGCUAGAAAUAGCAAGUUAAAAUAAGGCUAGUCCGUUAUCAACUUGAAAAAGUGGCACCGAGUCGGUGC2′Ome(U(ps)U(ps)U(ps)U_3’ |
| TRAC | 5’_2′OMe(G(ps)A(ps)G(ps))AAUCAAAAUCGGUGAAUGUUUUAGAGCUAGAAAUAGCAAGUUAAAAUAAGGCUAGUCCGUUAUCAACUUGAAAAAGUGGCACCGAGUCGGUGC2′OMe(U(ps)U(ps)U(ps)U_3' |

RNA; (ps) indicate phosphorothioate. Underlined bases denote the target sequence.

**Supplemental Table 2.** Details of conjugated antibodies used for flow cytometry.

| **Antigen** | **Clone** | **Fluorochrome** | **Manufacturer** | **Catalogue number** |
| --- | --- | --- | --- | --- |
| CD2 | RPA-2.10 | APC | BD Biosciences | 560642 |
| CD3 | UCHT1 | AF488 | BD Biosciences | 557694 |
| CD3 | SK7 | APC | eBioscience | 47-0036-42 |
| CD3 | UCHT1 | BV650 | BD Biosciences | 563852 |
| CD4 | RPA-T4 | APC-H7 | BD Biosciences | 560158 |
| CD4 | SK3 | BV510 | BD Biosciences | 562970 |
| CD8 | RPA-T8 | BV650 | BD Biosciences | 563821 |
| CD8 | RPA-T8 | BV510 | BD Biosciences | 563256 |
| CD34 | QBEnd10 | PE | Beckman Coulter | IM1250U |
| human CD45 | HI30 | BV786 | BD Biosciences | 563716 |
| mouse CD45 | 30-F11 | BV421 | BD Biosciences | 563890 |
| CD45RO | UCHL1 | BV711 | BioLegend | 304236 |
| CD45RA | HI100 | PerCP-Cy5.5 | BD Biosciences | 563429 |
| CD197/CCR7 | G043H7 | Alexa Fluor 647 | BioLegend | 353218 |
