# Supplementary material for: An “off-the-shelf” CD2 universal CAR-T therapy for T-cell malignancies
Source: Leukemia. 2023 Oct 5;37(12):2448–56. doi: 10.1038/s41375-023-02039-z (PMC10681896; doi:10.1038/s41375-023-02039-z)
Supplement: Supplementary file 2 — S1 [file 41375_2023_2039_MOESM2_ESM.pdf]

Target Information & Location - TRAC						GAGAATCAAAATCGGTGAAT	GUIDE-Seq Reads (TRAC)		
Target Site	Mismatch	Chr	Neares gene	Location	Distance from exon	Sequence Alignment	Double TRAC/CD2-1	Double TRAC/CD2-2	Double TRAC/CD2-3
chr14_22547575	0	chr14	TRAC	Exon 1	0	GAGAATCAAAATCGGTGAAT	10909	8799	13801
chr14_22547474	8	chr14	TRAC	Intergenic	-32	GAGGATCAGGGTTAGGACAT	40		
chr2_23593780	8	chr2	AC011239.2	Intergenic	-22719	ACAGCTCCAAGGCAGTGAAT	38	69	58
chr5_172347770	6	chr5	SH3PXD2B	Intronic	438	GAAAGGAAACATCTGTGAAG			4

Target Information & Location - CD2						ACAGCTGACAGGCTCGACAC	GUIDE-Seq Reads (CD2)		
Target Site	Mismatch	Chr	Nearest gene	Location	Distance from exon	Sequence Alignment	Double TRAC/CD2-1	Double TRAC/CD2-2	Double TRAC/CD2-3
chr1_116760608	0	chr1	CD2	Exon 3	0	ACAGCTGACAGGCTCGACAC	5782	4452	7052
chr5_74685250	8	chr5	HEXB	exon 1	0	ACAGCTCCATGGCCGCTCGG	1709	887	1285
chr16_49356400	7	chr16	AC007614.1	Intergenic	5896	ACAGCTCCAGGGGTGCCAGC	695	435	512
chr2_23593780	8	chr2	AC011239.2	Intergenic	-22719	ACAGCTCCAAGGCAGTGAAT	38	69	58
chr19_12939472	5	chr19	CALR	Exon 3	0	GTTGCTGAAAGGCTCGAAAC	19	6	
chr3_193835990	8	chr3	LINC02038	Intergenic	-6570	CCGACTGCCAGGTCAGACTA	588		
chr1_200990162	8	chr1	KIF21B	exon 20	0	ATCACCGACTGCCAGGCCAC		367	
chr16_49357294	8	chr16	AC007614.1	Intergenic	6790	AGAAGTCACCGCCTCATCAC			489
chr2_85788412	7	chr2	ATOH8	Intergenic	346	TTCGATGACTGGCTCTGTCAC			151
chr3_195785866	7	chr3	MUC4	Exon 2	0	ACAGGTGACACCA-CCCCTC			4
chr5_172347744	6	chr5	SH3PXD2B	Intergenic	412	ACCGCTGCCCTGGGTGACAC			4

Mismatched bases: A C T G
