# Supplementary figures and images for: An “off-the-shelf” CD2 universal CAR-T therapy for T-cell malignancies
Source: Leukemia. 2023 Oct 5;37(12):2448–56. doi: 10.1038/s41375-023-02039-z (PMC10681896; doi:10.1038/s41375-023-02039-z)

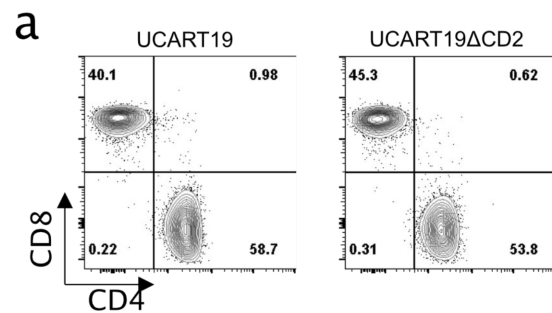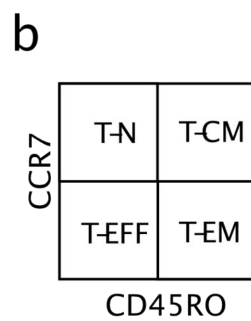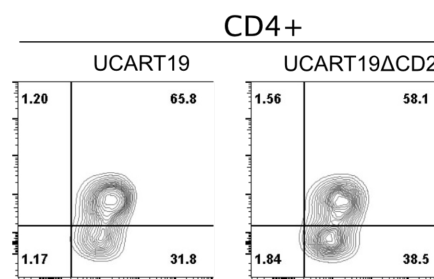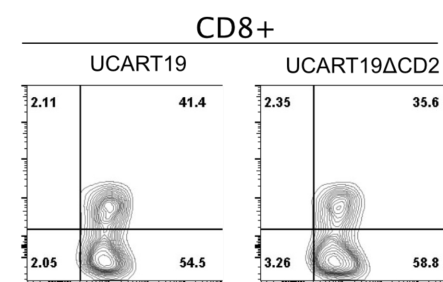

Supplement: Supplementary file 3 — S2 [file 41375_2023_2039_MOESM3_ESM.pdf]

a.

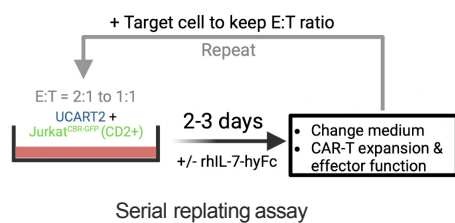

b.

## CAR-T cell expansion

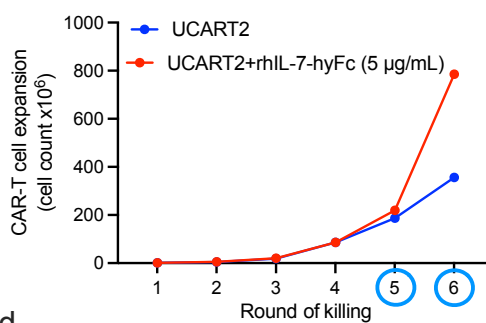

c.

## ⑤ 24 hr BLI killing curve

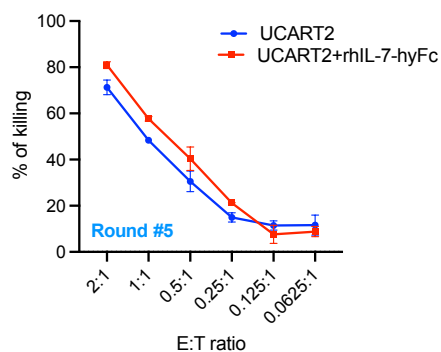

d.

## ⑥ 24 hr BLI killing curve

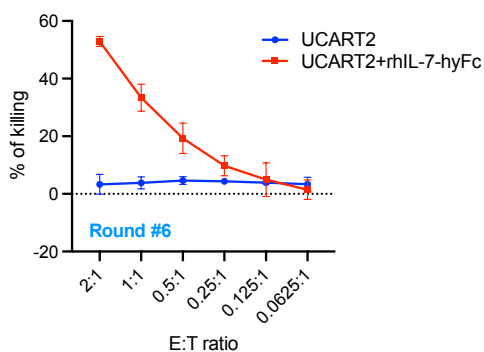

Supplement: Supplementary file 4 — S3 [file 41375_2023_2039_MOESM4_ESM.pdf]

a.

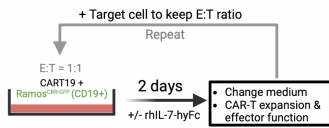

b.

CAR-T cell expansion

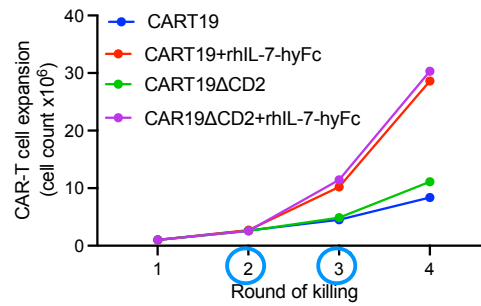

c.

②

24 hr killing curve

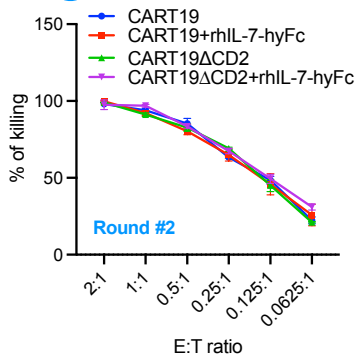

d.

③

24 hr killing curve

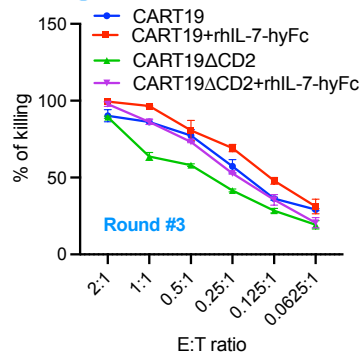

Supplement: Supplementary file 5 — S4 [file 41375_2023_2039_MOESM5_ESM.pdf]
