# Supplementary material for: An “off-the-shelf” CD2 universal CAR-T therapy for T-cell malignancies
Source: Leukemia. 2023 Oct 5;37(12):2448–56. doi: 10.1038/s41375-023-02039-z (PMC10681896; doi:10.1038/s41375-023-02039-z)
Supplement: Supplementary file 6 — Supplemental Methods [file 41375_2023_2039_MOESM6_ESM.docx]

**Supplemental Methods**

**Cell lines**

CD2 positive human T-ALL cell lines, MOLT-3 (ACC-84), and Jurkat (ACC-282) were obtained from the DSMZ-German Collection of Microorganisms and Cell Cultures (Leibniz, Germany). CD2 positive human cutaneous T cell lymphoma (CTCL) cell line HH (CRL-2105), B cell adult lymphoblastic leukemia cell line NALM6 (CRL-3273) and B cell lymphoma cell line Ramos (CRL-1596) were obtained from ATCC (Manassas, VA). HH and NALM6 cell lines were transduced with EF1α^CBR-GFP^ lentivirus to express Click Beetle Red (CBR) luciferase and GFP. Following transduction, the GFP-positive cells were sorted and cloned to establish the CBR-GFP cell lines (HH^CBR-GFP^ and NALM6^CBR-GFP^). All cell lines were mycoplasma tested periodically. The T-ALL patient-derived cell line, DCFI-15, was a kind gift from the Public Repository of Xenografts (PRoXe).

**CAR-T design**

The heavy and light chain sequences were obtained from Hybridoma OKT 11 (ATCC CRL-8027) via commercial sequencing (GenScript, Piscataway, NJ). To generate the CD2-28ζ construct, the single-chain variable fragment (scFv) DNA sequence was synthesized and cloned into the backbone of a 2^nd^ generation CAR with a CD28 co-stimulatory domain in the pLVM lentiviral vector (kindly provided by Dr. Carl June, U of Penn.). The CAR construct was modified to co-express a cytoplasmic truncated human CD34 via a P2A peptide to allow for the detection and purification of the CAR-T cells^1^. A CD19 targeting construct, CD19-28ζ, was generated for use as a non-targeting control as previously described^1^.

**Viral vector production**

To produce lentivirus, Lenti-X 293T cells (Takara Bio, Mountain View, CA) were transfected with CAR lentiviral vector and packaging plasmids, pMD.Lg/rRRE, pMD.G, and pRSV.REV, using Lipofectamine 2000 (Invitrogen, Waltham, MA), as per instructions. The virus was harvested 36 hours post-transfection and concentrated as previously described^1^.

**CRISPR/Cas9 gene editing and CAR-T production from human T-cells**

Human primary peripheral blood mononuclear cells (PBMCs) were acquired from de-identified donors at Mississippi Valley Regional Blood Center. As determined by Washington University’s HRPO (Human Research Protection Office) in 2009, these anonymous human blood cells are not considered human subject research. PBMCs were isolated using Ficoll-Paque PLUS density gradient media, density 1.077 g/mL (Cytiva, Cat# 17144003), as per instructions. T cells were cultured in Xcyte media supplemented with 10 ng/ml IL-2, and 10 ng/ml IL-15 in the presence of anti-CD3/CD28 beads (Bead to cell ratio 3:1, Thermo-Fisher Scientific Cat# 40203D). On day +2 post activation, CD3/CD28 beads were removed and 1×10^7^ T cells were electroporated in 100 μL MaxCyte buffer containing 15 μg spCas9 (TriLink Biotechnologies, San Diego, CA.) and 20 μg of each gRNA (IDT) using a MaxCyte GT (Gaithersburg, MD). Full guide sequences can be found in **Supplemental Table 1**. The cells were transduced with CD2-28ζ or CD19-28ζ lentivirus in the presence of polybrene (Sigma Aldrich, St Louis, MO) at the final concentration of 6 μg/ml on day +3. Transduced T-cells were then expanded for 6 days before being used in later experiments.

**Targeted deep sequencing**

The *CD2* locus was amplified with primers: forward: 5’-accaaagatctcctggacttgt-3’ and reverse: 5’-gcatgcctctgcctactgag-3’. The *TRAC* Locus was amplified with primers: forward: 5’-tggggcaaagagggaaatga-3’; reverse: 5’-gtcagatttgttgctccaggc-3’. PCR products were sequenced using the Illumina MiSeq platform (San Diego, CA). Editing efficiency was determined as a percentage of sequencing reads with indels aligned to reads obtained from WT cells.

**Chromium release assay**

CAR-T cells were incubated with the Jurkat, MOLT-3, and HH cell lines (1x10^4^ total cells/well) at an effector: target [E: T] ratio ranging from 25:1 to 0.25:1 in RPMI supplemented with 5% FBS. The chromium release assay was performed according to the instruction from the manufacturer (Perkin Elmer, Waltham, MA).

**FACS-based cytotoxicity assay**

Target cells, NALM6^CBR/GFP^, were seeded at a density of 25,000 cells per well in a 96-well V-bottom plate in R10 media (RMPI1 640, 10% FBS). Effector cells UCART19 and UCARTΔCD2 were co-cultured with target cells at E: T Ratios ranging from 2:1 – 1:16 and incubated at 37^o^C for 20 hrs. Absolute cell counts of viable target cells were quantified by flow cytometry using 7-aminoactinomycin D (7-AAD) and GFP. Data was collected using the Attune NxT flow cytometer and analyzed using FlowJo V10.

**Single-cell cytokine profiling of CAR-T**

CAR-T cells were labeled with Violet stain A according to the manufacturer's protocol (IsoPlexis), followed by incubation with target Ramos cell at a 1:2 Effector to Target (E: T) ratio for 20 hours. After stimulation, residual target cells were depleted with CD19 biotin microbeads using the Miltentyi AutoMACS according to the manufacturer's instructions. Cells were stained with Alexa Fluor 647-conjugated anti-human CD8 (IsoPlexis) at room temperature for 20 min and loaded onto an IsoCode chip. Each IsoCode chip contains ~12,000 microchambers pre-patterned with a full copy of the 32-plex antibody array (as shown in **Figure 3c**). The polyfunctional profile (2+ proteins per cell) of single cells was evaluated by IsoSpeak software version 2.7.0.0.

**Animal models**

Animal protocols were established in compliance with Washington University School of Medicine Institutional Animal Care and Use Committee (IACUC) regulations. NOD. Cg-*Prkdc*^scid^ *Il2rg*^tm1Wjl^/SzJ (NSG) male mice six to ten weeks old were used in all animal experiments.

**HH xenograft model**

To assess the anti-leukemic effect of the UCART2 in vivo, 5x10^5^ HH^CBR-GFP^ cells were inoculated into the NSG mice via tail vein injection on day -4. UCART2 or UCART19 controls (2x10^6^) were administered via the lateral tail vein on Day 0. In the UCART2/rhIL-7-hyFc combination experiment, HH^CBR-GFP^ tumor-bearing mice were administered with a sub-optimal dose of UCART2 or UCART19 (1x10^6^). rhIL-7-hyFc (provided by NeoImmuneTech Inc., Rockville, MD) was delivered via subcutaneous injection on days +1, +15, and +29. BLI (bioluminescence imaging) was performed in a blinded fashion weekly to assess the *in vivo* tumor burden, as previously described^2, 3^.

**NALM6^CBR-GFP^ xenograft model**

CD19+ NALM6^CBR-GFP^ cells were used as the target to evaluate the efficacy of UCART19 after CD2 deletion *in vivo*. 1x10^6^ NALM6^CBR-GFP^ cells were inoculated into the NSG mice via tail vein injection on day -5. UCART19Δ2 or UCART19 cells (1x10^6^) were I.V. infused on Day 0. Weekly BLI and survival were monitored, as described above.

**T-ALL patient-derived xenograft (PDX) model**

NSG mice were engrafted with 5x10^5^ DCFI-15 cells on day 0, followed by the I.V. infusion of 1x10^6^ UCART2 or UCART19 on day 12. Peripheral blood was analyzed bi-weekly to track the circulating CAR-T and tumor cells. Red blood cells were lysed using Red Blood Cell Lysing Buffer (Sigma-Aldrich) and washed with running buffer (PBS supplemented with 0.5% bovine serum albumin and 2 mM EDTA). Cells were prepared for flow cytometry analysis by resuspending the cells in the running buffer and a pre-titrated amount of fluorochrome-labeled monoclonal antibodies. Absolute circulating CAR-T cells (7-AAD negative, hCD34 positive) or tumor cells (7-AAD negative, hCD45 positive, hCD34 negative) were calculated using AccuCount Fluorescent Particles (SpheroTech, Cat#ACFP-100-3). Full detail of the antibodies can be found in **Supplemental Table 2**. Data were analyzed using FlowJo V10.

**Guide-Seq**

Off-target analysis with Guide-seq was performed as previously described^1^.

**Statistical analysis**

Sample size and data analysis for this study were determined following the general guideline for animal studies^4^. Time-to-death distributions were described using the Kaplan Meier product limit method and compared by log-rank test. All other in vivo data were summarized using means and standard deviations. The differences were compared using two-sample Student t-test, one-way ANOVA, or two-way ANOVA for repeated measurement data as appropriate, followed by post-hoc multiple comparisons for between-group differences of interest. Based on the law of diminishing returns, Mead et al. recommended that a degree of freedom (DF) of 10-20 associated with the error term in an ANOVA will be adequate for a pilot study to estimate preliminary information. The normality of data was assessed graphically using residuals and the similarity of variance across groups was also assessed visually by checking the estimated variance of each group. A logarithm transformation was performed as necessary to better satisfy the normality and homoscedasticity assumptions. The resultant p-values were adjusted by a step-down Bonferroni adjustment for multiple comparisons if needed. Compared to the widely used Bonferroni adjustment, a step-down method is more powerful (smaller adjusted p-values) while maintaining strong control of the familywise error rate. All analyses were two-sided and significance was set at a p-value of 0.05. The statistical analyses were performed using GraphPad Prism 9 (San Diego, CA) and SAS 9.4 (SAS Institutes, Cary, NC).

**References:**

1. Cooper ML, Choi J, Staser K, Ritchey JK, Devenport JM, Eckardt K*, et al.* An "off-the-shelf" fratricide-resistant CAR-T for the treatment of T cell hematologic malignancies. *Leukemia* 2018 Sep; **32**(9)**:** 1970-1983.

2. DiPersio JF, Staser K, Cooper M. Immunotherapy for T-Cell ALL and T-Cell NHL. *Clin Lymphoma Myeloma Leuk* 2020 Sep; **20 Suppl 1:** S56-S58.

3. Kim MY, Jayasinghe R, Devenport JM, Ritchey JK, Rettig MP, O'Neal J*, et al.* A long-acting interleukin-7, rhIL-7-hyFc, enhances CAR T cell expansion, persistence, and anti-tumor activity. *Nat Commun* 2022 Jun 13; **13**(1)**:** 3296.

4. Festing MF, Altman DG. Guidelines for the design and statistical analysis of experiments using laboratory animals. *ILAR J* 2002; **43**(4)**:** 244-258.
