# Supplementary material for: An “off-the-shelf” CD2 universal CAR-T therapy for T-cell malignancies
Source: Leukemia. 2023 Oct 5;37(12):2448–56. doi: 10.1038/s41375-023-02039-z (PMC10681896; doi:10.1038/s41375-023-02039-z)
Supplement: Supplementary file 7 — Supplemental figure legend [file 41375_2023_2039_MOESM7_ESM.docx]

**Supplemental Figure 1. Guide-seq analysis for off-target activity in UCART2.** ***Top*)** Sites of off-target activity aligned to TRAC gRNA target sequence. ***Bottom*)** Sites of off-target activity aligned to CD2 gRNA target sequence.

**Supplemental Figure 2.** **Immunophenotype and subsets of UCART19 and UCART19ΔCD2 by flow cytometry analysis.** **a.** CD4 and CD8 subsets in UCART19 and UCART19ΔCD2; **b.** Immunophenotype of UCART19 and UCART19ΔCD2, defined by CD45RO and CCR7 surface expression. T-N: naïve T cells; T-EFF: effector T cells; T-EM: effector memory T cells; T-CM: central memory T cells.

**Supplemental Figure 3. rhIL-7-hyFc enhances UCART2 expansion and effector function. a.** Experimental design: UCART2 cells were challenged with CD2+ target cell Jurkat^CBR-GFP^ at E:T ratio of 2:1 to 1:1 every 2-3 days, in the presence or absence of rhIL-7-hyFc (5 μg/mL); **b.** CAR-T cell expansion was monitored by flow cytometry; **c-d**. CAR-T cytotoxic function was measured by BLI killing assay (24 hr) after 5 or 6 rounds of replating.

**Supplemental Figure 4. rhIL-7-hyFc rescues reduced effector CAR-T function due to CD2 deletion.** **a.** Experimental design: CART19 or CART19ΔCD2 cells were challenged with CD19+ target cell Ramos^CBR-GFP^ at 1:1 E:T ratio every 2 days, in the presence or absence of rhIL-7-hyFc (5 μg/mL); **b.** CAR-T cell expansion was monitored by flow cytometry; **c-d.** CAR-T cytotoxic function was measured by BLI killing assay (24 hr) after 2 or 3 rounds of replating.
